# Molecular and clinicopathological analysis of three cases of gastric juvenile polyposis

**DOI:** 10.1002/jgh3.12781

**Published:** 2022-06-28

**Authors:** Yuya Yamashiro, Yuka Yanai, Tsutomu Takeda, Takuo Hayashi, Yoichi Akazawa, Noboru Yatagai, Hiroya Ueyama, Hidetaka Eguchi, Akihito Nagahara, Takashi Yao, Tsuyoshi Saito

**Affiliations:** ^1^ Department of Human Pathology Juntendo University School of Medicine Tokyo Japan; ^2^ Department of Gastroenterology Juntendo University School of Medicine Tokyo Japan; ^3^ Intractable Disease Research Center Juntendo University, Graduate School of Medicine Tokyo Japan

**Keywords:** *BMPR1A*, juvenile polyposis, loss of heterozygosity, *SMAD4*, *TP53*

## Abstract

**Background and Aim:**

Juvenile polyposis (JP) is a rare disease known to be associated with mutations either in *SMAD4*/*BMPR1A*. JP is known to often develop into malignant tumors, with a reported probability of 9–50%. However, the mechanisms of its carcinogenesis are not fully understood. We tried to elucidate the mechanisms of malignant transformation underlying this condition in three cases of gastric JP.

**Methods:**

We selected polyps from each patient displaying varying degrees of atypia and their nearby normal polyps and compared them using immunohistochemistry, Sanger sequencing, and loss of heterozygosity (LOH) analysis of *SMAD4*, *BMPR1A*, and *TP53.*

**Results:**

Two of the three cases were suspected of having germline *SMAD4* mutations based on their familial medical histories; the remaining case was found to have a *SMAD4* germline mutation following preoperative genetic testing. All three cases were shown to present with both SMAD4 positive and negative areas across each lesion, with the neoplastic lesions tending to show stronger nuclear SMAD4 expression. This expression was closely associated with the *SMAD4* LOH status; however, we also noted paradoxical SMAD4 expression in the neoplastic lesions despite the biallelic inactivation of *SMAD4* revealed in the genetic evaluation.

**Conclusions:**

These data suggest that strong nuclear expression of SMAD4, even when seemingly paradoxical, seems to be closely associated with dysplastic polyps in JP. Complete inactivation of *SMAD4* was not shown to be essential for the development of dysplastic polyps in gastric JP, and other pathways seemed to be involved in the acquisition of the malignant phenotype.

## Graphical abstract

Strong nuclear expression of SMAD4, even under the presence of genetic alterations, seems to be closely associated with dysplastic polyps in juvenile polyposis (JP). Complete inactivation of SMAD4 was not shown to be essential for the development of dysplastic polyps in gastric JP, and other pathways seemed to be involved in the acquisition of the malignant phenotype.

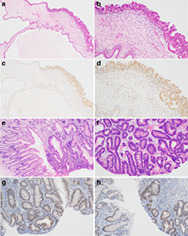

## Introduction

Juvenile polyposis (JP) is a rare autosomal dominant inherited disease with an incidence of 1 in 100 000 individuals per year.^1^ The clinical criteria for diagnosis can be summarized as follows: (i) five or more juvenile polyps in the colon; (ii) multiple juvenile polyps in two or more organs of the gastrointestinal tract; and (iii) a family history of JP and juvenile polyps in the gastrointestinal tract.^2^ Genetic diagnosis is also an important consideration, and 45–55% of patients are known to have germline mutations in *SMAD4* or *BMPR1A*.^3^ Both *SMAD4* and *BMPR1A* belong to the transforming growth factor‐β (TGF‐β) superfamily; however, patients with *SMAD4* and *BMPR1A* mutations are known to present with different clinical presentations. Patients with *SMAD4* mutations are reported to experience a higher prevalence of gastric polyps and twice the frequency of anemia as that of patients with *BMPR1A* mutations.^3^ In addition, JP is known to often develop into malignant tumors, with a reported probability of 9–50%.^3^ SMAD4 is a signal transducer of TGF‐β, which acts as a cell growth suppressor, and it is assumed that these *SMAD4* mutations prevent TGF‐β mediated cellular repression, resulting in the transformation of normal cells into cancerous cells or the appearance of a more malignant phenotype.^4^ However, it is not fully understood which molecular mechanisms are involved in this transformation and malignancy, which is characterized by numerous benign hamartomatous polyps. Here we describe three cases of JP in which total gastrectomy was performed. In addition, we selected multiple polyps displaying varying degrees of atypia, including those with carcinomatous changes from these patients and investigated the genetic abnormalities possibly accumulated during this neoplastic change. This kind of study focusing on the genetic and immunohistochemical differences between gastric multiple polyps from the same patient has not previously been performed, and would provide new insight to the understanding of occurrence of dysplastic change in JP.

## Materials and methods

### Histopathological evaluations

We evaluated the pathological features for each sample and then defined specific features based on their degree of atypia. High‐grade dysplasia (HGD) was defined as presumed neoplasia with enlarged nuclei, atypia, and increased chromatin. Low‐grade dysplasia (LGD) was defined as disordered adenomatous round to spindle‐shaped nuclei, and indefinite for neoplasia (IDN) was defined as mildly enlarged nuclei and increased chromatin, but without the disordered phenotype of the HGD and LGD lesions. Those areas of hyperplasia within the glandular epithelium without atypia and the normal gastric mucosa were referred to as foveolar hyperplasia (FH). We used each of these atypical features and their associations with juvenile polyps or FH as the evaluation set in all subsequent investigations. This study was reviewed and approved by the Juntendo University School of Medicine Institutional Review Board (#2016107).

### Immunohistochemistry

Immunohistochemical analyses were performed on 4 μm thick formalin‐fixed, paraffin‐embedded tissue sections and antibodies against SMAD4 (1:50, Santa Cruz Biotechnology, Dallas, TX, USA), BMPR1A (1:200, Abcam, Cambridge, UK), and p53 (Predilute, BioGenex, Fremont, CA, USA). The immunohistochemistry (IHC) results were then evaluated by two pathologists (T.S. and Y.Y.). SMAD4 and BMPR1A were evaluated using nuclear staining^5^, ^6^ and cytoplasmic staining,^6^ respectively. IHC score was calculated by counting both the percent positive cells and the intensity of staining as follows: 1 × (% of tumor cells with 1 + signal intensity) + 2 × (% of tumor cells with 2 + signal intensity) + 3 × (% of tumor cells with 3 + signal intensity). p53 IHC was performed as previously reported^7^ and staining was judged as overexpression when the number of positive cells accounted for >10% of the total cell count.

### DNA extraction

DNA was extracted from the formalin‐fixed paraffin‐embedded (FFPE) samples using the QIAamp FFPE tissue kit (Qiagen, Antwerp, Belgium), and each tumoral component (HGD, LGD, and IDN) was microdissected to prevent contamination by non‐tumoral tissue.

### Sanger sequencing

Mutations in the *SMAD4*, *BMPR1A*, and *TP53* genes were evaluated by Sanger sequencing using a series of primer pairs similar to those listed on the Cancer Hotspot Panel (Table S1, Supporting information).

### Loss of heterozygosity analysis

The loss of heterozygosity (LOH) analysis at the *SMAD4*, *BMPR1A*, and *TP53* loci was performed for each of the lesions across all three cases using six (D18S845, D18S1110, D18S474, D18S69, D18S74E, and D18S851), three (D10S1687, D10S1744, and D10S573),^5^, ^8^ and two markers (AFM‐238WF2 and TP53), respectively^9^ (Table S1). Each sample exhibiting an allelic imbalance factor >1.5 or <0.5 for at least one of these markers was considered to be demonstrating LOH.

### Next‐generation sequencing

Case 1 was also evaluated using the Ion Ampliseq Cancer Hotspot Panel v2 (Thermo Fisher Scientific Waltham, MA, USA), with these evaluations focusing on six specific lesions demonstrating varying degrees of atypia. The details of these evaluations were described in a series of previous publications.^5^, ^7^, ^9^, ^10^

## Case presentations

### Case 1

**Figure 1:**
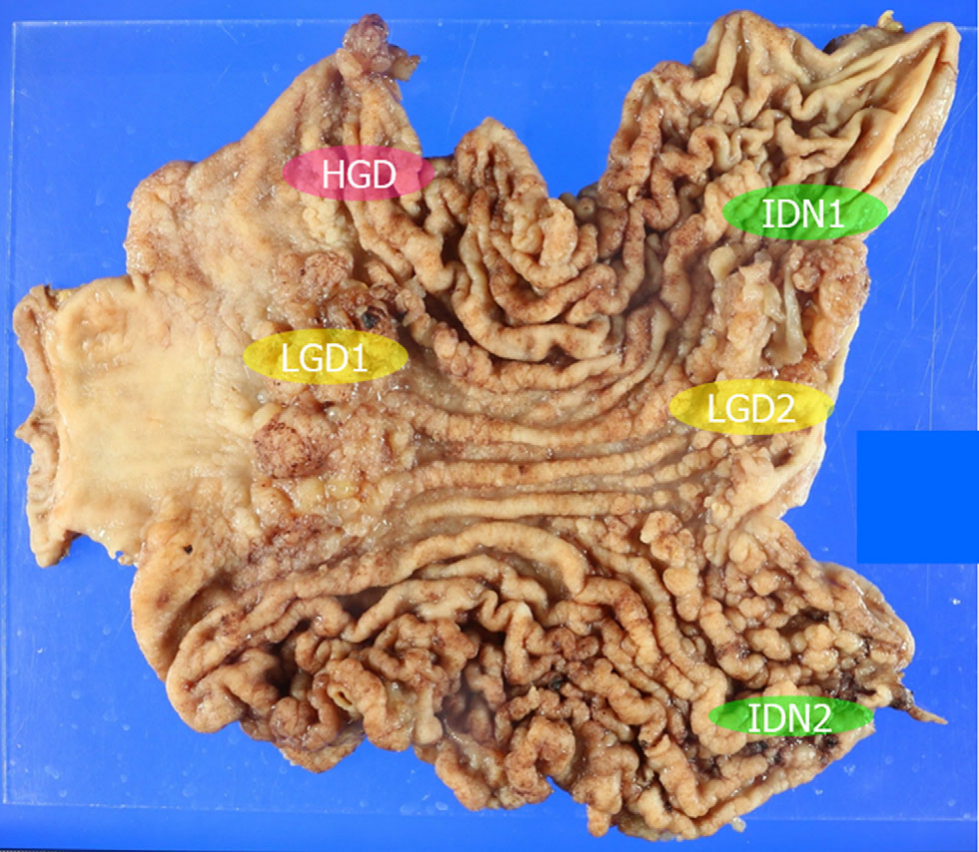
Gross findings from the resected stomach tissues of Case 1. Histological examination of the whole sections identified one HGD, two LGD, and two IDN lesions. HGD, high‐grade dysplasia; IDN, indefinite for neoplasia; LGD, low‐grade dysplasia.

**Figure 2:**
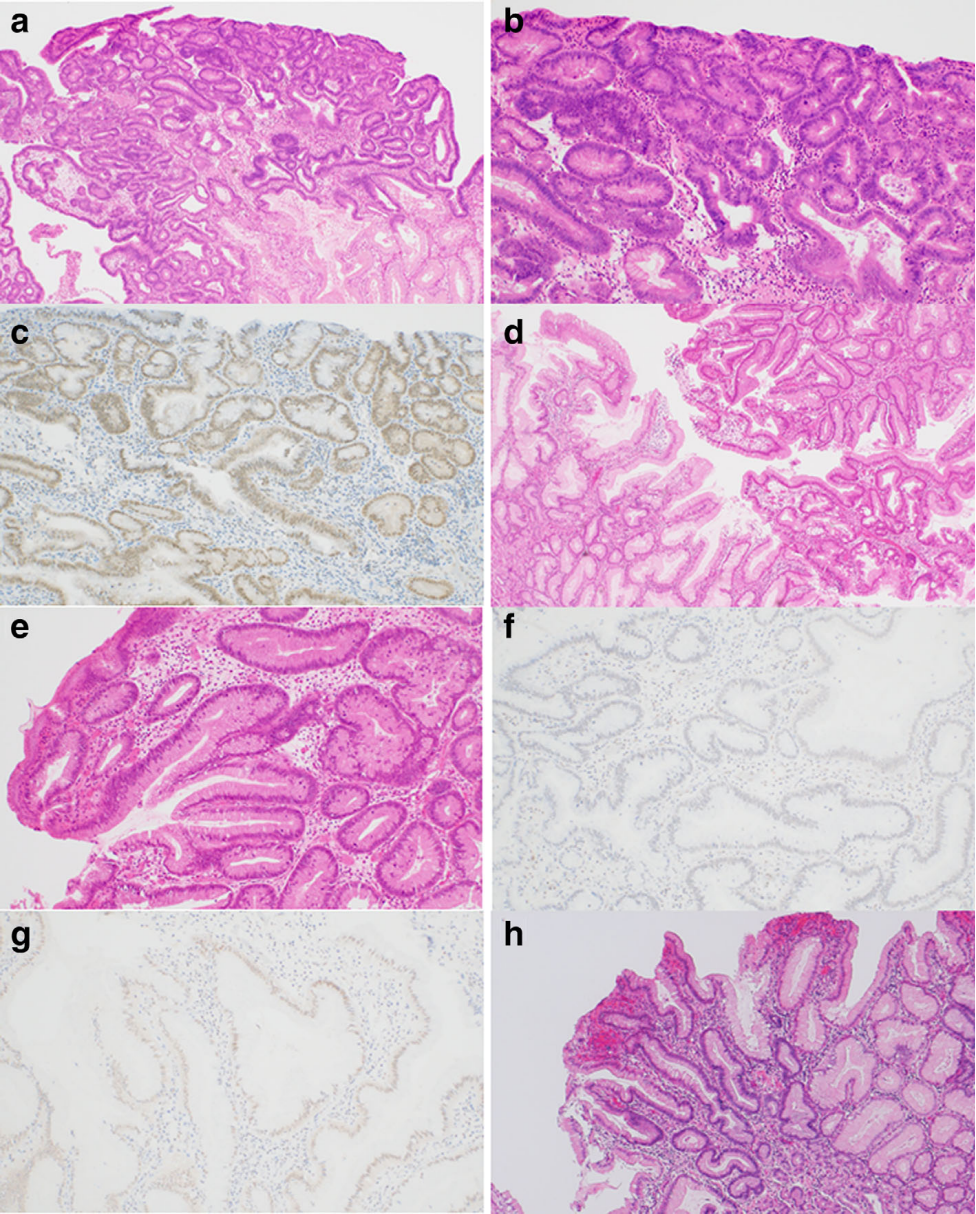
Histological features for Case 1. HGD lesion shows complex glandular structures with enlarged atypical nuclei within a background of hyperplastic foveolar epithelium (a: ×40, b: ×100). (c) SMAD4 IHC shows strong and diffuse nuclear staining across the HGD lesion (×100). (d,e) LGD2 lesion from Case 1 (d: Right side, and e) shows nuclear atypia usually associated with neoplasticism but it is less severe than that of the HGD lesion. Left side in d shows a JP area (d: ×40, e: ×100). F: LGD2 lesion exhibits a loss of SMAD4 expression (×100). (g) JP nearby LGD2 lesion shows weak nuclear staining for SMAD4 and was called as negative (×100). (h) The IDN1 lesion from Case 1 could not be classified as neoplastic, but it did present with enlarged nuclei. HGD, high‐grade dysplasia; IDN, indefinite for neoplasia; IHC, immunohistochemistry; JP, juvenile polyp; LGD, low‐grade dysplasia.

A 48‐year‐old woman underwent esophagogastroduodenoscopy (EGD) after a positive finding of occult blood in her fecal samples collected during a routine medical checkup. Multiple polyps were observed in the stomach, and the patient was referred to Juntendo Univ. hospital. No particular symptoms were observed, and other than a history of sinusitis surgery, no other medical history was noted. Her older sister (Case 2) was also diagnosed with multiple polyps in the stomach on EGD, and her father died of rectal cancer 45 years prior. He had 12 brothers and sisters, and at least 3 had a history of colon polyps, colon cancer, and gastric cancer. EGD showed multiple erythematous soft polyps from the fornix to the antrum of the stomach. There were no obvious lesions associated with other invasive cancers, and a small number of polyps were also found in the duodenum. Multiple biopsies of the gastric polyps showed hyperplasia of the glandular epithelium, edema, and inflammatory cell infiltration in the stroma, which were consistent with JP, although there were no obvious histological signs of neoplastic changes. The duodenal polyps presented with inflammatory cell infiltration of the epithelium, and two polyps in the descending colon were endoscopically resected and diagnosed as tubular adenomas. Blood tests revealed no anemia with 11.5 g/dL hemoglobin (Hb) and a slightly reduced albumin level at 3.9 g/dL. The biopsy was followed by total gastrectomy, and the resected stomach is shown in Figure 1. We identified numerous polyps throughout the stomach, especially in the cardia and lower body to the antrum, which tended to be tall and large. The entire area of the resected specimen was then examined, with these evaluations reporting that most of these polyps were composed of non‐dysplastic epithelial hyperplasia, stromal edema, and inflammatory cell infiltration. In addition, we identified one HGD lesion, two LGD lesions, and one IDN lesion, which were then subject to further evaluations (Fig. 2). The results of the genetic analysis and IHC are shown in Table 1. The HGD lesions were SMAD4 positive, but there was no LOH in these samples or their surrounding polyps. The LGD1 lesion demonstrated SMAD4 expression, but the surrounding polyps and non‐neoplastic area were negative for this protein, and LOH was detected in both the LGD1 and surrounding polyp areas. The LGD2 lesion and its associated polyps were negative for SMAD4, and both demonstrated LOH at the *SMAD4* locus. The two IDN lesions were almost SMAD4 positive, but no LOH was detected. Mutation analysis of the *SMAD4* gene by Sanger sequencing revealed no pathogenic alterations, and all of the lesions were positive for BMPR1A with no signs of LOH for this locus. *BMPR1A* mutation analysis identified a c.4C>A and c.1343‐11T>C mutation at all sites, which are both known to be benign polymorphisms.^11^ *TP53* did not demonstrate either LOH, overexpression, or genetic alterations in these samples. In addition, although both the HGD and LGD1 lesions were evaluated by next‐generation sequencing (NGS), no significant genetic abnormalities were detected.

**Table 1:** Results of genetic analysis and IHC

	SMAD4			BMPR1A			TP53			
	LOH	IHC score	Target sequence	LOH	IHC score	Target sequence	LOH	IHC overexpression	Target sequence	NGS
Case1										
Normal		200	—		200	c.4C>A, c.1343‐11T>C		(−)	(−)	—
HGD	(−)	300	—	(−)	300	c.4C>A, c.1343‐11T>C	(−)	(−)	(−)	—
JPs	(−)	200	—	(−)	100	c.4C>A, c.1343‐11T>C	(−)	(−)	(−)	—
LGD1	(+)	300	—	(−)	200	c.4C>A, c.1343‐11T>C	(−)	(−)	(−)	—
JPs	(+)	60	—	(−)	100	c.4C>A, c.1343‐11T>C	(−)	(−)	(−)	—
LGD2	(+)	0	—	(−)	200	c.4C>A, c.1343‐11T>C	(−)	(−)	(−)	N.A.
JPs	(+)	100	—	(−)	100	c.4C>A, c.1343‐11T>C	(−)	(−)	(−)	N.A.
IDN1	(−)	240	—	(−)	300	c.4C>A, c.1343‐11T>C	(−)	(−)	(−)	N.A.
JPs	(−)	100	—	(−)	200	c.4C>A, c.1343‐11T>C	(−)	(−)	(−)	N.A.
IDN2	(−)	200	—	(−)	300	c.4C>A, c.1343‐11T>C	(−)	(−)	(−)	N.A.
JPs	(−)	100	—	(−)	100	c.4C>A, c.1343‐11T>C	(−)	(−)	(−)	N.A.
Case2										
Normal		150	—		100	c.4C>A, c.1343‐11T>C		(−)	(−)	
LGD 1	(−)	300	—	(−)	300	c.4C>A, c.1343‐11T>C	(−)	(−)	(−)	
JPs	(−)	100	—	(−)	100	c.4C>A, c.1343‐11T>C	(−)	(−)	(−)	
LGD 2	(−)	260	—	(−)	300	c.4C>A, c.1343‐11T>C	(−)	(−)	(−)	
JPs	(−)	100	—	(−)	200	c.4C>A, c.1343‐11T>C	(−)	(−)	(−)	
LGD 3	(−)	300	—	(−)	300	c.4C>A, c.1343‐11T>C	(−)	(−)	(−)	
JPs	(−)	100	—	(−)	200	c.4C>A, c.1343‐11T>C	(−)	(−)	(−)	
IDN	(−)	300	—	(−)	300	c.4C>A, c.1343‐11T>C	(−)	(−)	(−)	
FH	(−)	100	—	(−)	150	c.4C>A, c.1343‐11T>C	(−)	(−)	(−)	
Case3										
Normal		100	—		200	c.4C>A, c.1343‐11T>C		(−)	(−)	
HGD	(+)	300	—	(−)	300	c.4C>A, c.1343‐11T>C	(−)	(−)	(−)	
JPs	(+)	0	—	(−)	100	c.4C>A, c.1343‐11T>C+c.1094G>A	(−)	(−)	(−)	
LGD1	(+)	200	—	(−)	300	c.4C>A, c.1343‐11T>C+c.871‐1G>C	(−)	(−)	(−)	
JPs	(+)	200	—	(−)	200	c.4C>A, c.1343‐11T>C	(−)	(−)	(−)	
LGD2	(+)	50	—	(−)	200	c.4C>A, c.1343‐11T>C+c.1166+3G>A	(−)	(−)	(−)	
JPs	(+)	0	—	(−)	100	c.4C>A, c.1343‐11T>C	(−)	(−)	(−)	
LGD3	(+)	150	—	(−)	300	c.4C>A, c.1343‐11T>C	(−)	(−)	(−)	
JPs	(+)	0	—	(−)	100	c.4C>A, c.1343‐11T>C+c.1066C>T	(−)	(−)	(−)	
FH	(−)	150	—	(−)	300	c.4C>A, c.1343‐11T>C	(−)	(−)	(−)	
HGD^†^	(+)	0	—	(−)	300	c.4C>A, c.1343‐11T>C	(−)	(−)	(−)	
IDN^†^	(+)	0	c.1579delA	(−)	300	c.4C>A, c.1343‐11T>C	(−)	(−)	(−)	
JPs^†^	(+)	0	—	(−)	300	c.4C>A, c.1343‐11T>C	(−)	(−)	(−)	

^†^ Duodenal lesion.

IHC, immunohistochemistry; LOH, loss of heterozygosity; N.A., not available.

### Case 2

**Figure 3:**
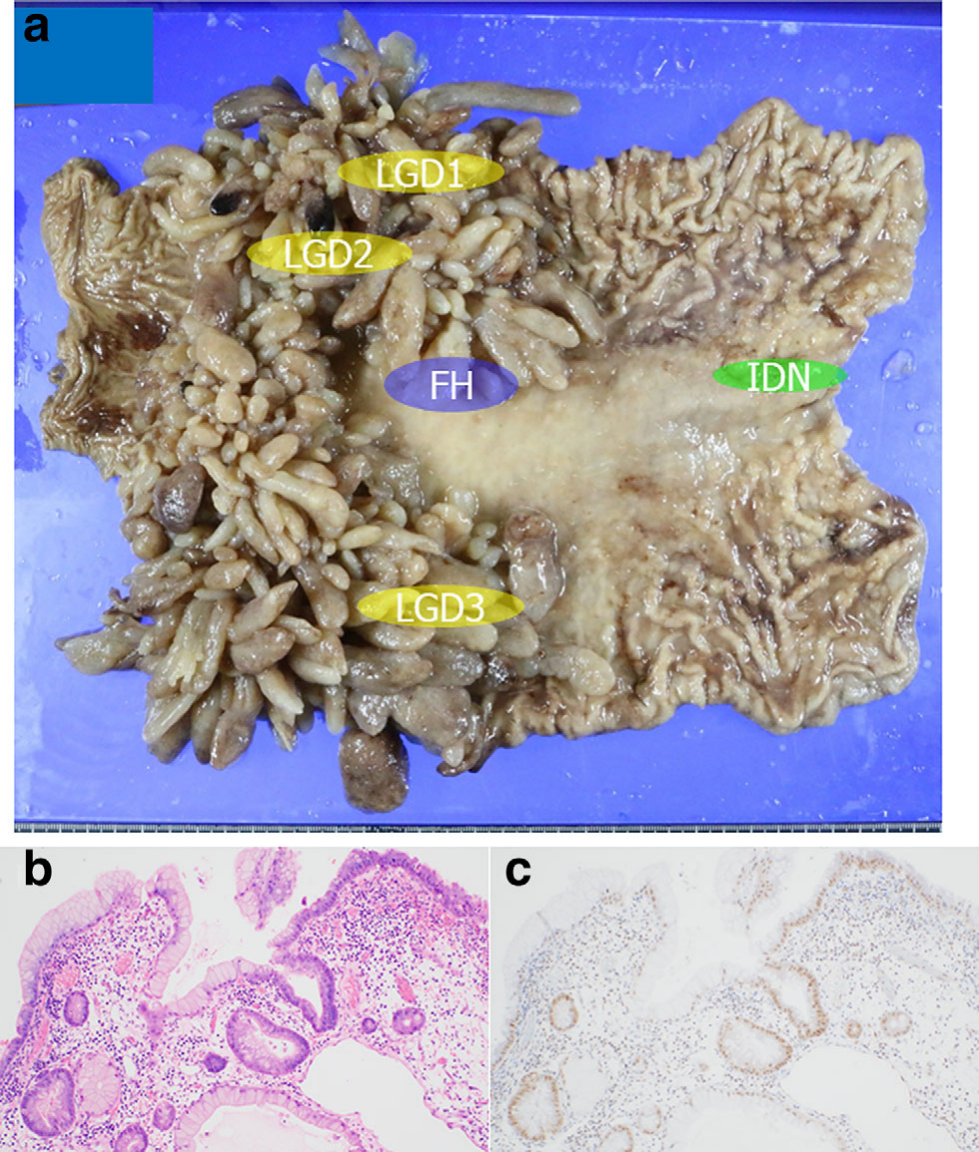
(a) Gross findings from the resected stomach tissues of Case 2 revealed multiple polyps in the stomach, especially in the lower body and antrum. (b) Atypical glands in the LGD3 lesion are admixed with JP (×100). (c) SMAD4 IHC reveals strong nuclear expression in the atypical glands and relatively weak nuclear expression in the JP (×100). IHC, immunohistochemistry; JP, juvenile polyp.

A 54‐year‐old woman was diagnosed with multiple polyps in the stomach by endoscopy and had been taking iron supplements for anemia. Her younger sister (Case 1) was diagnosed with JP and underwent surgery at Juntendo Univ. hospital; thus, she was referred to our hospital. Blood tests revealed anemia at 6.5 g/dL Hb and reduced albumin levels at 3.0 g/dL, and endoscopic examination identified multiple large polyps in the stomach, especially in the lower body and antrum. We also resected two polyps from the cecum, with both cecal polyps demonstrating dilated glands and stromal edema with inflammatory cell infiltration, consistent with the features of juvenile polyps. Total gastrectomy was performed following the JP diagnosis, and these resected tissues are shown in Figure 3a. We identified and evaluated three LGD and one IDN lesion in this sample as well as one region of FH. IHC for SMAD4 revealed that all lesions were positive for SMAD4 expression, whereas most areas within the juvenile polyps were positively stained, but there were also scattered areas of negative staining. LOH at the *SMAD4* locus was not detected across all of the lesions, but BMPR1A expression was preserved in all of these samples. We also noted that this case presented with the same *BMPR1A* mutations as Case 1 (Table 1), and we also recorded no significant findings for *TP53*.

### Case 3

**Figure 4:**
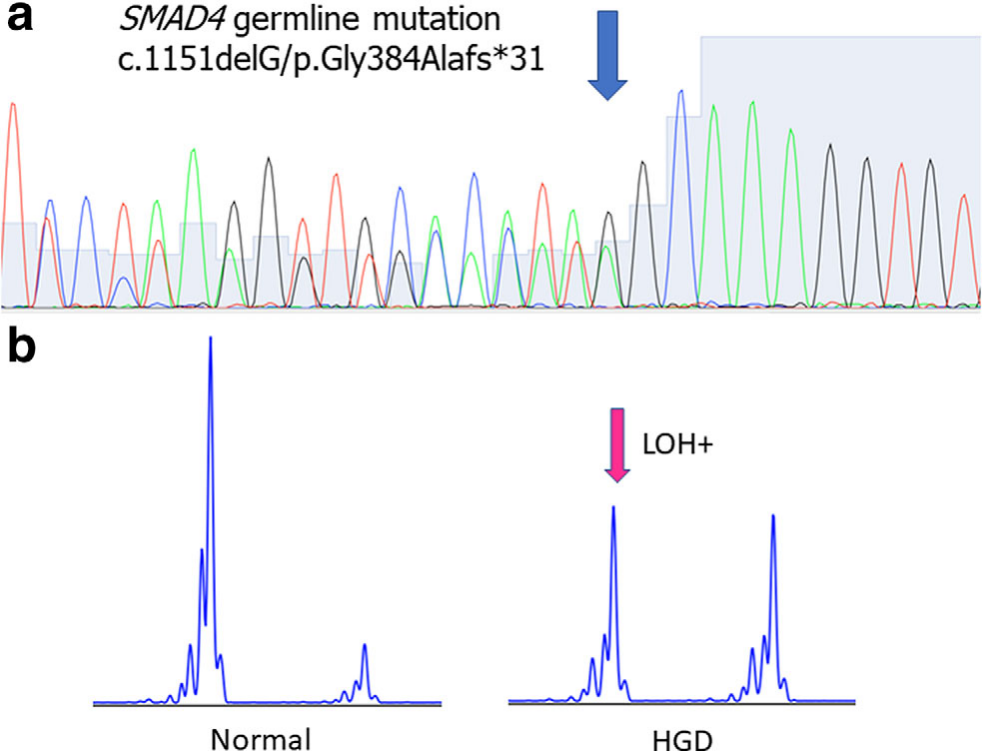
(a) Case 3 underwent genetic testing prior to resection, which revealed that this patient had experienced a germline deletion in exon 10 of *SMAD4* (c.1151delG/p.Gly384Alafs*31). (b) The results of the *SMAD4* LOH analysis (marker D18S1110) of the HGD lesion from Case 3. HGD, high‐grade dysplasia; LOH, loss of heterozygosity.

**Figure 5:**
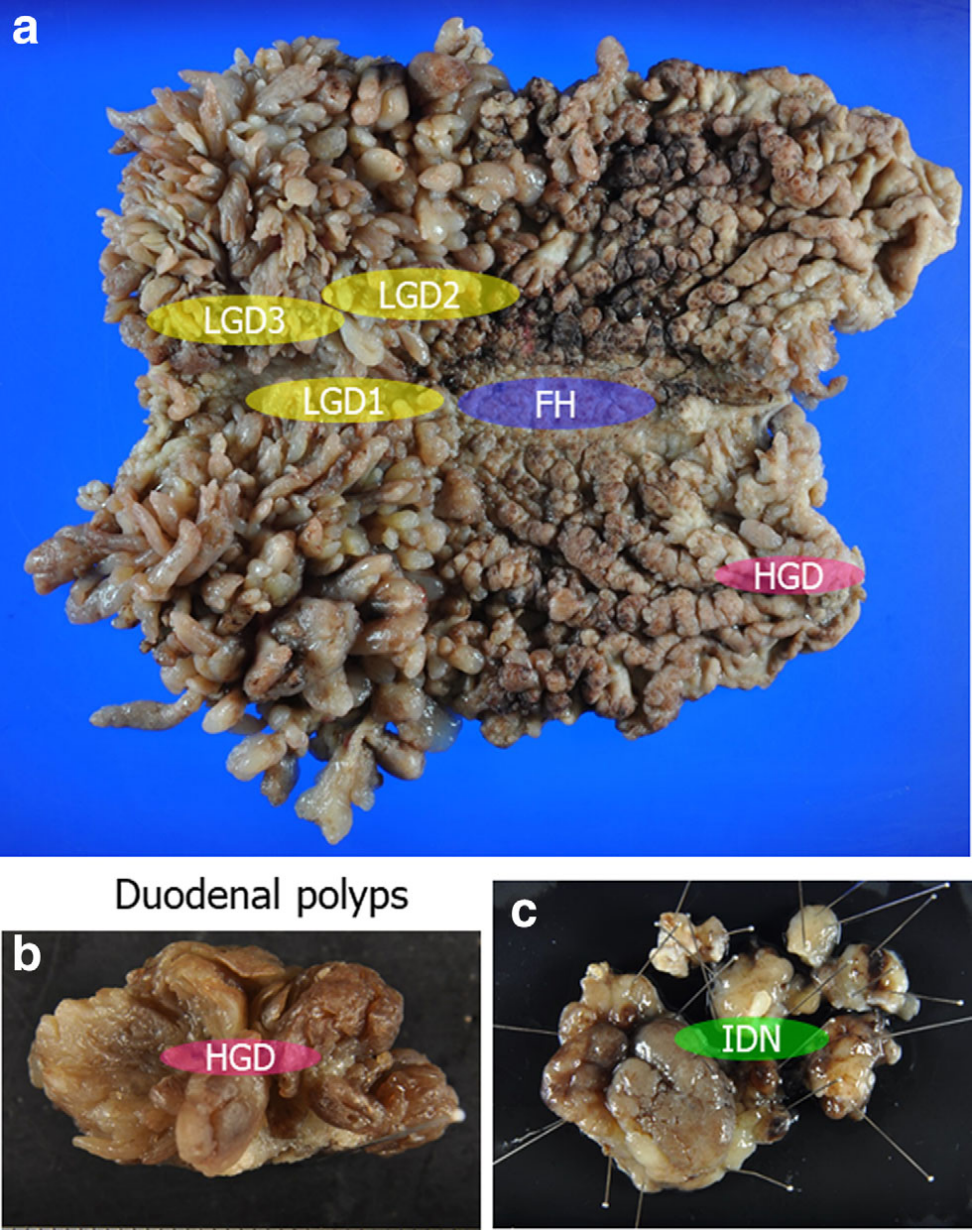
(a) Gross findings for the resected stomach tissues from Case 3 revealed multiple polyps in the stomach. (b,c) Duodenal polyps with an HGD (b) and IDN lesion (c). HGD, high‐grade dysplasia; IDN, indefinite for neoplasia.

**Figure 6:**
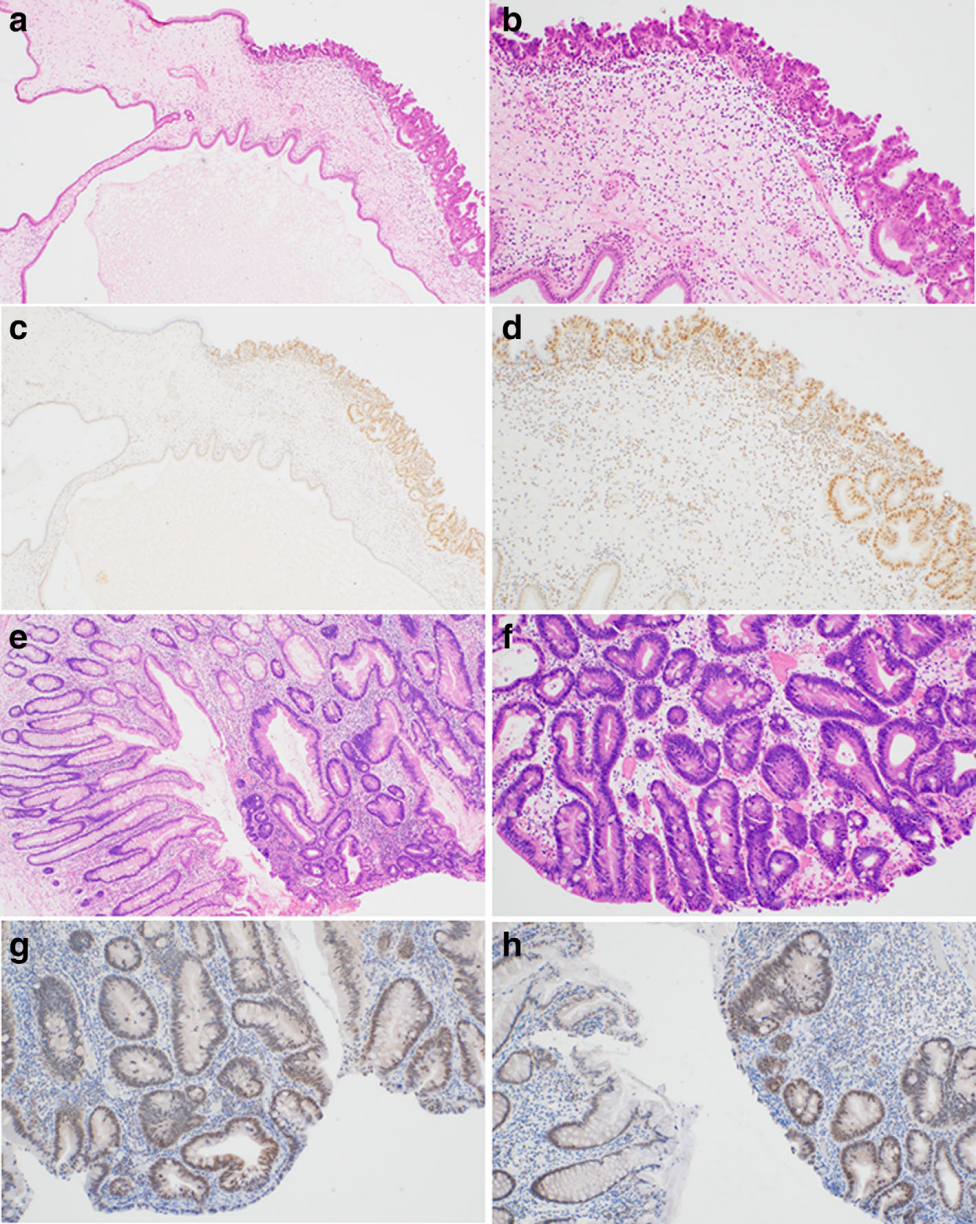
Histological features from Case 3. (a,b) The HGD lesion is composed of atypical glands with structural atypia and clear margins (a: ×40, b: ×100). (c,d) SMAD4 IHC reveals strong nuclear expression in the HGD lesion but the surrounding JP was negative for SMAD4 expression (c: ×40, d: ×100). (e,f) The LGD1 lesion is comprised of atypical glands with clear margins and mild structural atypia (e: ×40, f: ×100). (g,h) SMAD4 IHC reveals moderate nuclear expression within the LGD lesion (g) and surrounding JP (left side in h) (g: ×40, h: ×100). HGD, high‐grade dysplasia; IHC, immunohistochemistry; JP, juvenile polyp; LGD, low‐grade dysplasia.

This patient had experienced frequent abdominal discomfort from her early and eventually underwent EGD at 47 years of age. EGD had multiple polyps in her stomach, and blood tests revealed that she was suffering from hypoproteinemia. She was diagnosed with Cronkhite–Canada syndrome and treated with steroids, but there was no improvement, and she was referred to our hospital when she was 52 years old. Both her mother and younger sister had been diagnosed with multiple polyps in the stomach and had undergone total gastrectomy at another hospital. EGD showed multiple polyps across the entire stomach and duodenum, some of which were erythematous. Biopsy revealed hyperplastic changes in the epithelium, edema, and inflammatory cell infiltration in the stroma, and colonoscopy revealed polyps in the cecum. Blood tests showed mild anemia with 10.3 g/dL Hb and low albumin levels at 2.5 g/dL. In addition, albumin scintigraphy revealed protein leakage from the stomach. Preoperative genetic testing confirmed a germline mutation in *SMAD4* (c.1151delG/p.Gly384Alafs*31, Fig. 4a), and total gastrectomy was performed following her diagnosis with JP. The cecal polyps were also resected. The gross appearance of the resected tissue revealed that the entire stomach was covered with numerous polyps making it difficult to identify the normal mucosa. One HGD, three LGDs, and one FH lesion were selected for our downstream evaluations, as shown in Figure 5a. This patient has subsequently undergone periodic endoscopic polyp resection of the duodenum, which allowed us to identify a further HGD and IDN lesion from these samples, which were then included in our evaluations (Fig. 5b,c). This patient experienced larger SMAD4 negative areas than the other two cases, but their HGD lesions demonstrated strong nuclear expression of this protein, despite the fact that the nearby polyps were negative for SMAD4. A total of two out of the three LGD lesions showed SMAD4 expression; however, there were many negative areas surrounding the LGD lesions (Fig. 6). LOH was observed at each of the investigated sites with the exception of the FH and HGD lesions, as shown in Figure 4b. Genetic analysis revealed a c.1579delA/p.I527Ffs mutation in the IDN lesion of the duodenum, which has not been previously reported but is unlikely to affect protein expression. Genetic mutation analysis also revealed several single‐nucleotide variants in the *BPR1A* gene including intronic mutations whose clinical significance is unknown (Table 1). We also observed the same mutations in this gene as those described in both Cases 1 and 2. These mutations are reported to have unknown clinical significance or have not been previously reported. However, our data suggest that these mutations are not associated with the loss of BMPR1A expression, when evaluated by IHC. There were no abnormalities in TP53 expression in this sample either.

## Discussion

Here we describe three cases of gastric JP. Patients in Cases 1 and 2 also had a small number of duodenal and colon polyps; however, their predominant lesions were found in the stomach. Case 3 showed multiple juvenile polyps in the duodenum and colon, suggesting a generalized gastrointestinal type. Cases 1 and 2 are siblings, and there was an extensive family history of gastrointestinal tract cancer within their family, suggesting the presence of several germline mutations. In addition, there is a high incidence of gastric polyposis in patients with *SMAD4* mutations^3^ and frequent epistaxis in Case 1 and her relatives, which may indicate hereditary hemorrhagic telangiectasia, suggesting a high probability of *SMAD4* germline mutations. Our analysis of the *SMAD4* mutations in these patients using the same primer pairs as those used in the Cancer Hotspot Panel did not identify any germline mutations; however, we are currently discussing whether patients should be tested for germline mutations in genetic counseling. We did identify a germline *SMAD4* deletion (c.1151delG/p.Gly384Alafs*31) in Case 3, and although similar mutations have not been reported in the past, other deletions and point mutations in exon 10 of the *SMAD4* gene have been reported.^12^ Cases 2 and 3 presented with anemia, which is consistent with the high prevalence of anemia in patients with *SMAD4* mutations.^3^ *SMAD4* acts as a tumor suppressor gene, and IHC against SMAD4 is known to be a useful and reliable ancillary tool for the evaluation of a patient's pathogenic predisposition to JP. Immunohistochemical abnormalities in SMAD4, decreased or loss of expression in the epithelium, are observed only in cases with genetic mutations in *SMAD4*.^13^ However, the role of *SMAD4* in polyp formation and its involvement in carcinogenesis has not been fully elucidated. It was reported that 9 out of 20 JPs with known *SMAD4* germline alterations presented with reduction or loss of SMAD4 nuclear protein expression in the epithelium, and five of those nine cases demonstrated *SMAD4* LOH, and two presented with a somatic stop codon mutation, indicating a correlation between protein expression and biallelic loss of function mutations.^14^ In contrast, none of the 12 polyps with *SMAD4* germline 4 bp‐deletion, including the 8 demonstrating reduced SMAD4 nuclear expression, presented with any known somatic mutations.^8^ Our data reveal a concordance between the presence of LOH and decreased SMAD4 IHC in Cases 1 and 2, suggesting that there is at least a minor association between protein expression and gene status. However, polyps in the stomach showed a variety of staining patterns including preserved, reduced, and loss of expression, and it seems that protein expression status is not essential for polyp formation. Furthermore, there are also relatively few studies supporting the significance of *SMAD4* in JP oncogenesis. Previous reports have shown that the SMAD4 protein expression does not correlate with the presence of atypia,^14^ and our data suggest that the degree of dysplasia did not necessarily correlate with the SMAD4 expression status of specific lesions. In fact, both HGD lesions from the stomachs of Cases 1 and 3 patients were positive for SMAD4 (both IHC score: 300), whereas the HGD lesions from the stomach of Case 3 patient showed strong SMAD4 expression despite LOH at the *SMAD4* locus and a significant germline *SMAD4* mutation. Interestingly, two of the LGD lesions (LGD1 in Case 1 [IHC score: 300] and LGD3 in Case 3 [IHC score: 150]) were positive for SMAD4 presenting with clear borders, whereas their neighboring polyps showed only focal and weak SMAD4 expression (Case 1) or did not exhibit any SMAD4 expression (Case 3). The reason for these paradoxical expression patterns is unknown; however, this phenomenon was also reported in a previous study.^13^, ^14^ These findings are not consistent with the classical Knudson's “two‐hit hypothesis,”^15^ and this hypothesis does not fit well with polyp formation and carcinogenesis in JP. It is in fact more likely that other oncogenic drivers may be involved in the tumorigenesis of JP. In addition, although previous studies have suggested that mutations in *TP53* may be associated with JP oncogenesis,^14^ another study failed to identify *TP53*, *KRAS*, and *BRAF* mutations by hot spot mutation analysis,^8^ being consistent with our findings. The use of comprehensive NGS, such as whole‐genome sequencing, is expected to shed more light on this question in the future.

As limitations of this study, we studied only three cases of JP, and three cases are too small to draw any definite conclusion. Furthermore, *SMAD4* germline mutation has not been clearly proved for two of those cases. In addition, NGS to seek other pathways involved in the dysplastic change of juvenile polyp could be performed in only few polyps due to sample quality problem, and NGS employed in this study was a small hot spot panel.

In conclusion, we selected 33 lesions from three JP specimens and evaluated their *SMAD4*, *BMPR1A*, and *TP53* genetic status and protein expression patterns. Our data suggest that strong nuclear SMAD4 expression, although often paradoxical, seems to be associated with neoplastic changes in JP. Complete inactivation of *SMAD4* does not appear to be essential for the development of dysplastic polyps in gastric JP, and other pathways other than *TP53* are likely involved in the acquisition of the malignant phenotype of gastric JP.

## Consent for Publication

Written consent to publish this information was obtained from all three patients.

## Supporting information


**Table S1.** Sequences of primers and microsatellite markers for SMAD4, BMPR1A and TP53.

## Data Availability

All data generated or analyzed during this study are included in this published article and Table S1.
